# P-1585. The Impact of MRSA Nasal PCR on Antibiotic De-escalation

**DOI:** 10.1093/ofid/ofae631.1752

**Published:** 2025-01-29

**Authors:** Adam Silverman, Pranisha Gautam-Goyal, Shivanthidevi Gandhi, Michael Oppenheim, Guillermo Ramirez, Gabriel Karkenny, Rubab Sohail

**Affiliations:** Northwell Health, Manhasset, New York; Zucker School of Medicine at Hofstra/Northwell, Manhasset, New York; North Shore University Hospital, New Hyde Park, New York; Northwell Health, Manhasset, New York; Zucker School of Medicine at Hofstra/Northwell, Manhasset, New York; Northwell Health, Manhasset, New York; Northwell Health, Manhasset, New York

## Abstract

**Background:**

The MRSA nasal PCR is a screening tool that may help decrease the use of antimicrobials. The MRSA PCR as a screening test has shown a high negative predictive value associated with various infections - including pneumonia, intra-abdominal infections, wounds, and bacteremia. Despite this, MRSA PCR has been under-utilized for the purposes of deescalating antibiotics targeting MRSA.

Odds Ratios

**Figure:**
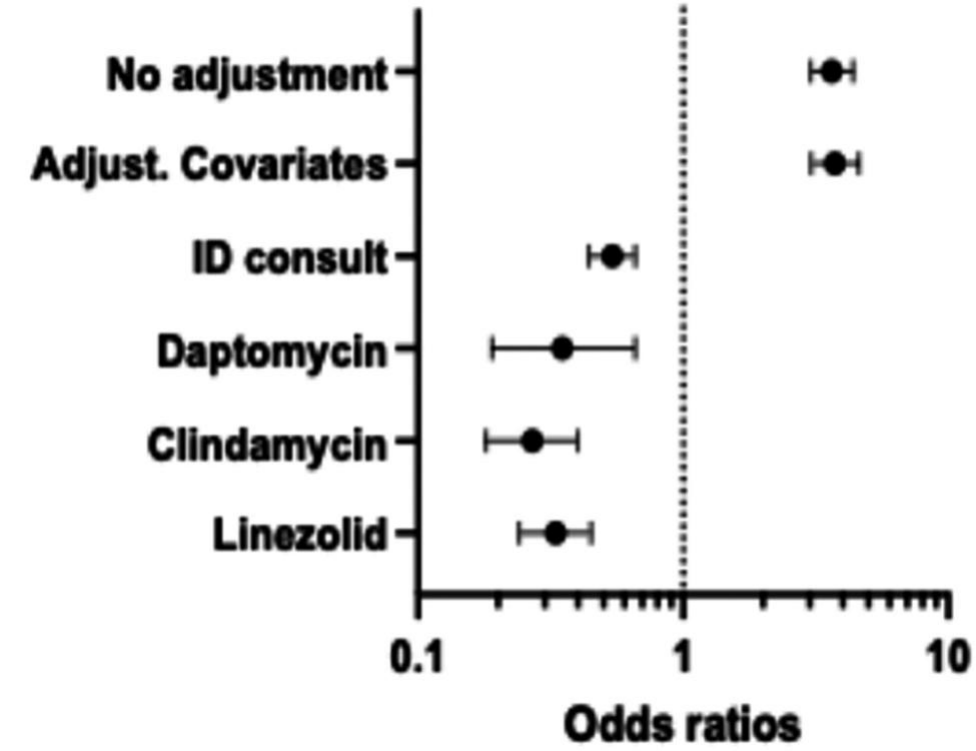

**Methods:**

This was a retrospective, observational, single-center cohort study within the Northwell Health system, approved by the Institutional Review Board. Data was collected from the electronic medical record between January 1, 2020, to December 1, 2021. We included adults admitted for inpatient care who had a MRSA PCR performed during the admission and were receiving antimicrobial therapy directed at treating MRSA. Clinical and demographic data were collected, in addition to the type of anti-MRSA therapy the patient was receiving, date the MRSA PCR resulted, date the anti-MRSA therapy was discontinued, and total days of anti-MRSA therapy. The primary objective was to determine if a negative MRSA PCR led to the discontinuation of anti-MRSA therapy within 48 hours of the result. Secondary objectives included the total days of anti-MRSA therapy, acute kidney injury (AKI) as discharge diagnosis, and hospital length of stay (LOS).

**Results:**

Of the 5,047 patients included for analysis, 89% (n = 4505) had a negative MRSA PCR result. The odds of anti-MRSA therapy discontinuation were 264% greater among subjects with a negative PCR compared to subjects with a positive PCR result (OR: 3.64, 95% CI: 3.03 – 4.37, p< 0.0001). After adjusting for covariates, this observation remained statistically significant, with the odds of anti-MRSA therapy discontinuation being 274% greater among subjects with a negative PCR compared to subjects with a positive PCR result (OR: 3.74, 95% CI: 3.04 – 4.59, p< 0.0001).

**Conclusion:**

A negative MRSA PCR result was significantly associated with anti-MRSA therapy discontinuation within 48 hours, both before and after adjusting for covariates. MRSA PCR is being increasingly recognized as a powerful antimicrobial stewardship tool and should continue to be utilized to help guide clinical decision making.

**Disclosures:**

**All Authors**: No reported disclosures

